# Study on the Geometric Design of Supports for Overhanging Structures Fabricated by Selective Laser Melting

**DOI:** 10.3390/ma12010027

**Published:** 2018-12-21

**Authors:** Kaifei Zhang, Guang Fu, Peng Zhang, Zhibo Ma, Zhongfa Mao, David Z. Zhang

**Affiliations:** 1 State Key Laboratory of Mechanical Transmissions, Chongqing University, Chongqing 400044, China; kaifeizhang@cqu.edu.cn (K.Z.); guangfu@cqu.edu.cn (G.F.); pengzhang@cqu.edu.cn (P.Z.); 20160701014@cqu.edu.cn (Z.M.); 2 Department of Mechatronics Engineering, College of Engineering, Shantou University, 243 Daxue Road, Shantou 515063, China; zfmao@stu.edu.cn; 3 College of Engineering, Mathematics and Physical Sciences, University of Exeter, North Park Road, Exeter EX4 4QF, UK

**Keywords:** support design, overhang, selective laser melting, Taguchi method, deformation, roughness

## Abstract

Additional structures are usually adopted to support the overhanging structures in order to resist the deformation of parts. Improper geometric design of the support structures may result in a sharp deterioration in the surface quality and a failure of manufacture, which affects the expansion in the use of selective laser melting (SLM) technology. In this research, cuboids were added into the conventional block support for a better heat dissipation. The Taguchi method was used to analyze the effect of the geometric design of this support on the part’s deformation and surface roughness. It was found that solid pieces or cuboids as support structures can reduce the deformation. However, their effects are weaker than those of teeth structures which decrease the deformation by more reliable connections. It is interesting that narrowing the gap between the cuboids and overhang can weaken the strength of teeth structures and then increases the deformation of part. In general, the distance between every two adjacent walls of support and the gap between the cuboids and the overhang have the greatest influence on the part’s deformation and surface quality respectively.

## 1. Introduction

Selective laser melting (SLM), a member of additive manufacturing (AM) technologies, adopts a laser beam to melt metal powders layer-by-layer based on the two-dimensional (2D) slice profile of a part in an inert gas environment. Due to the complete melting of the raw metal powders, it allows the rapid production of fully dense and complicated parts within a single process, with mechanical performances exceeding the conventional material specifications [1]. After years of research and development, it has gradually matured in the application in the areas of aerospace, automobile, mold, and healthcare industries. However, there are still some inherent shortcomings that need to be overcome in SLM technology, such as the poor surface roughness of as-built part and the high probability of component deformation.

Staircase effect is an important factor leading to poor surface quality of selective laser melted (SLMed) part [2]. It can be alleviated by decreasing the layer thickness or the polar angle of surface to improve the surface roughness, which is ascribed to the stepped approximation by layers of inclined and curved surfaces [3]. Meanwhile, selecting the optimal build orientation of part to decrease the quantity of overhanging structures can also effectively optimize the surface roughness. On the other hand, a large amount of incompletely melted powders sticking to the surfaces of part also lead to the bad surface quality. In addition to the stair step effect, a new mathematical model that includes the presence of particles on top surfaces was presented for an accurate prediction of the surface roughness [4]. In order to reduce the powder sticking effect, the researches on the optimization of process parameters, such as scan speed and laser power, were taken place by [5,6]. A decrease in scan speed or an increase in laser power which will provide a higher energy density can improve the surface quality in the horizontal plane while a contradictory effect can be obtained in the vertical plane [7]. The re-melting parameters are also significant for achieving a better morphology of surface [8].

In the SLM process, large thermal stresses within a solidified part are usually induced by the nonuniform temperature distribution due to the rapid melting and solidification of the powder [9]. As a result, it will cause a deformation of the part, which will then affect the final part’s quality and even lead to an interruption of the building process. Unidirectional and alternating laser scan strategies were compared by thermo-mechanical simulations in their effects on the temperature distribution in order to understand the generation of residual stress and then control the deformation of the fabricated part [10]. Optimizing the process parameters, especially the volume energy density of the laser beam, was considered to improve the SLM machinability of the difficult-to-process overhanging structure [11]. In addition, several other effective methods have been proposed, such as using an intermediate powder mixture, controlling building chamber temperature and increasing the preheating temperature to effectively reduce the thermal deformation of fabricated part caused by the high temperature gradient [1,12].

**Figure 1:**
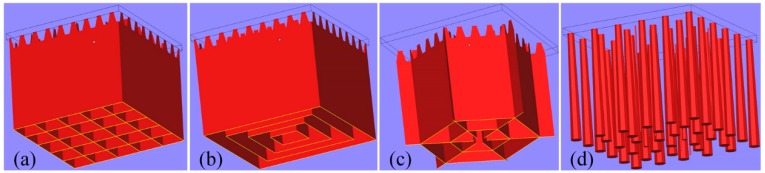
Conventional designs of support structures: (**a**) block support; (**b**) contour support; (**c**) web support; and (**d**) cone support.

**Figure 2:**
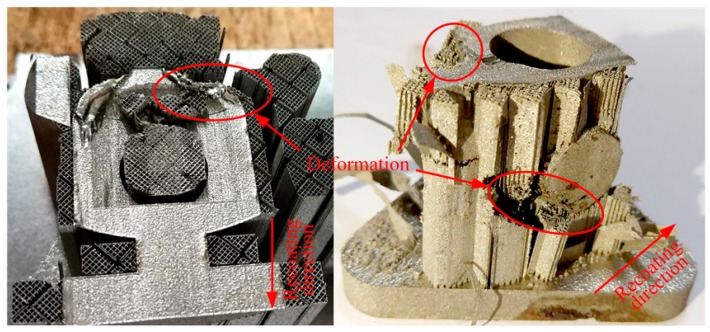
A failed part due to the improper geometric design of support structures.

For a better prevention of deformation, several types of support structures (Figure 1) are also commonly used to help form complex-shaped parts with overhanging structures in SLM. Except for the above conventional support structures, several other design methods of innovative support structures had been proposed in [13,14,15]. Nevertheless, superfluous support structures will result in larger time consumption, labor intensity, material waste, and poor surface quality of parts. Therefore, adapting the selective laser sintering (SLS) material principles to SLM was considered as an original method of support structures elimination [16]. However, its application scope is limited. Because the pulsed laser can achieve the structures with a higher porosity and a quicker scanning speed, it was employed to fabricate support structures to reduce the labor intensity and enhance the production efficiency [17]. According to [18,19], topology optimization method and optimizing the part orientation are also two useful methods to cut down the quantity of support structures. However, a deficiency or an improper geometric design of support structures may result in a failure of component’s manufacturing as shown in Figure 2. Consequently, the significance of the support structure parameters should also be given enough attention [20]. For example, in the design of the unconventional ‘Y’ support structure [15], the non-uniformly spaced struts created larger deformation of the supported thin plate than the uniformly spaced struts of the ‘IY’ support structure and smaller spacing between support struts lead to a better surface profile.

**Figure 3:**
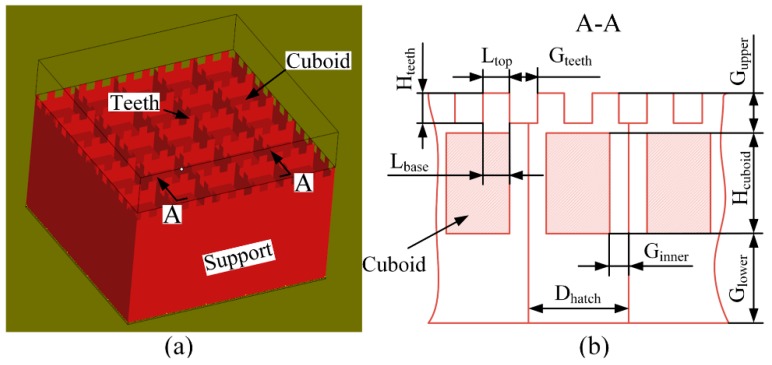
Block support structure with cuboids for heat dissipation: (**a**) three dimensional shape and (**b**) A-A local cross section sketch.

In the meantime, the influence of the support structure on heat dissipation has received more and more attention. A method of adding heat balance support was used to improve parts accuracy in selective laser sintering [21]. The quicker heat conduction of support structures makes the development of finer grains in the boundary areas where the support structures and the specimen are separated [22]. Therefore, when optimizing its geometric design by simulations, the support structure should also be taken into consideration with the solid part [23]. According to [24], the overhang deformation can also be reduced through including a solid piece as a heat sink under the overhang. In this study, an unconventional block support structure, whose interspaces are fully or partially filled by solid cuboids for heat dissipation (Figure 3), was used to anchor the designed specimens with some overhanging structures. The effects of the geometric parameters of this support on the deformations and the bottom surface qualities of the overhanging structures of the specimens were analyzed by the Taguchi method.

## 2. Solid Piece as Support Structure for Heat Dissipation

**Figure 4:**
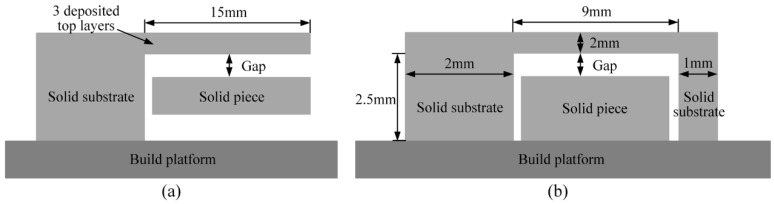
Different deformation evaluation models in (**a**) previous simulation study and (**b**) experiment study.

In addition to anchoring the AMed component to the build platform, the support structures also change the heat dissipation condition during the processing of the component. In [24], a finite element approach was introduced to analyze the thermo-mechanical responses in the electron beam additive manufacturing (EBAM) process of overhanging structures. As depicted in Figure 4a, a solid piece as a heat sink was added beneath the overhang and its effect on the temperature induced deformation was evaluated by using a 2D thermo-mechanical model. The overhang thickness was kept at three deposited layers (0.07 mm layer thickness) and the gap was set at three levels as 0 mm, 0.63 mm, and 6.3 mm. Moreover, the processing of the overhang without a solid piece was also simulated and their results of deformation magnitudes were compared together. It was found that the overhang area still has a much larger distortion than the solid area, although there is a solid piece inserted beneath the deposited top layers to improve the heat dissipation. However, a considerable deformation drop can be observed based on the deformation information of the top surface from simulations. In addition, the maximum deformation magnitude came down with the decrease of the gap between the overhang and the solid piece. It reflected that using a solid piece under the overhang may effectively decrease the overhang deformation. Unfortunately, this finding was lack of experimental demonstration.

**Figure 5:**
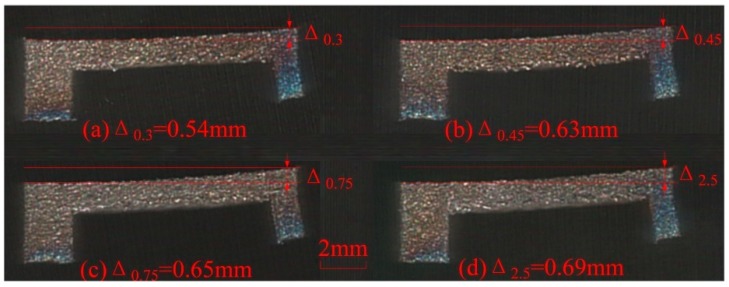
Deformations of specimens with different gaps of (**a**) 0.30 mm; (**b**) 0.45 mm; (**c**) 0.75 mm; and (**d**) 2.5 mm.

The SLM process is similar to EBAM since they are both powder bed AM technologies. They can produce parts with comparable microstructures, relative densities, and static properties in tension [25]. Their major difference is located in the used energy source. Therefore, the similar solid piece as support structure is inserted under the designed overhanging structure (Figure 4b) in the SLM process. The gap in Figure 4b was also set at three levels as 0.30 mm, 0.45 mm, and 0.75 mm. The specimens are formed by SLM under the same process parameters and a specimen without solid piece beneath it is also fabricated for comparison. After SLM processing, the 1 mm solid substrates of the specimens are cut firstly by wire electrical discharge machining. Therefore, the residual stresses existed in the specimens are released suddenly to cause deformations of overhangs at different levels as shown in Figure 5. When the gap size between the solid piece and the overhang increases from 0.30 mm to 0.45 mm and then to 0.75 mm, the deformation magnitude (Δ) goes up from 0.54 mm to 0.63 mm and then to 0.65 mm. In contrast, the deformation magnitude achieves 0.69 mm since there is no solid piece under the overhang. It is demonstrated that the deformation magnitudes of the specimens with solid pieces have decreased comparing to that of the specimen without solid piece. As the total energy input remains unchanged, the inserted solid in powder bed, with higher conductivity and larger density, can improve the heat dissipation condition in the same heat influenced region. Therefore, it would contribute to lower the top surface temperature and the powder bed temperature [24]. On the other hand, it can be found that the effect of the solid piece on the deformation of overhang would weaken with the increase of the gap between the solid piece and the overhang. The maximum deformation magnitudes are cut down by only 0.06 mm (Gap = 0.45 mm) and 0.04 mm (Gap = 0.75 mm) while it is cut down by 0.15 mm when the gap is 0.30 mm. In consequence, it is necessary to select an optimized gap distance so as to effectively reduce the deformation without being fused with the overhang part.

Although the inserted solid piece can minish the deformation of part in some extent, this contact-less support is still not be sufficient to anchor the overhang with longer length or larger bottom surface. Therefore, it is proposed to insert solid cuboids to the conventional block support structure to enhance the heat dissipation performance of support in this research. In the following, the effect of the geometric design of this new support structure on the distortion and the bottom surface quality of overhang is studied by the Taguchi method.

## 3. Experimental Methods

### 3.1. Equipment and Material

In this study, the commercial SLM equipment EOSINT M280 (EOS GmbH, Krailling, Germany) was used to perform the experiments. Its energy source is a single-mode ytterbium fiber laser which is operating at a continuous wave with wavelength of 1070 nm. The energy intensity distribution of laser beam is a Gaussian profile. The gas-atomized Titanium alloy Ti–6Al–4V powder (EOS GmbH, Krailling, Germany) was used as the feedstock material and its chemical composition is detailed listed in Table 1.

The process parameters of solid parts were also particularly summarized in Table 2. The contours of the part were irradiated by the same scanning speed and laser power as those building the core of the part. In addition, according to other researches [26,27], the scanning strategy significantly influenced the part’s temperature distribution in the SLM processing. For better understanding the difference of the bottom surfaces’ profiles of the overhanging structures with different heights, the parallel-hatching scanning strategy was employed whereby the scanning lines in all layers are parallel to the *X*-axis, i.e., the recoating direction. From the study in [28], it can be concluded that the improper scan length also greatly affects the temperature distribution of the SLMed part to cause a larger deformation. In order to easily distinguish the grades of deformations, the scan length in this work was set as infinitely long, that is to say, any part would not be scanned by partition in all layers. The cuboids for heat dissipation in the block support as depicted in Figure 3 were formed by the process parameters of solid parts while the thin walls of support were manufactured by 80 W power and 400 mm/s scanning speed. The block supports including cuboids and thin walls were exposed every two layers, which is different from the processing mode (layer by layer) of solid parts.

### 3.2. Experimental Design

#### 3.2.1. Design of Specimens

**Figure 6:**
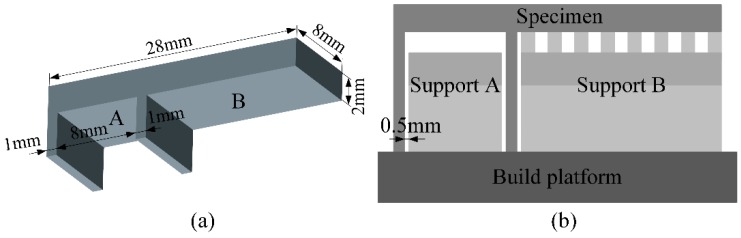
(**a**) 3D model of used specimens and (**b**) specimen with different supports.

As depicted in Figure 6a, a simple specimen with two kinds of overhanging structures was designed to study the effects of different support structures on the SLMed specimens’ qualities. The overhang A (Figure 6a) fixed on two sides and supported by support A (Figure 6b) without teeth structures was used to investigate the influence of support structures on the roughness of its bottom surface. The overhang B (Figure 6a) anchored at only one end and supported by support B (Figure 6b) was used to analyze the influence of geometric design of support structures on the distortion of SLMed parts. The support A and the support B were both block support with cuboids for heat dissipation as shown in Figure 3. Because the overhang A without any supports had been proven to be manufacturable by using SLM technology according to some previous experimental works, the teeth of the support A were deleted to prevent their effects on the final quality of the bottom surface of the overhang A. According to the research in [29], the residual stress is also determined by the overhang thickness and it becomes serious with increasing the overhang thickness. In our work, the overhang thickness (Figure 6a) is set as 2 mm. In order to be removed easily from the building platform by wire electrical discharge machining, all the specimens were built on the additional bases with height of 0.5 mm.

#### 3.2.2. The Taguchi Method

An experimental test was performed to find values that enable achieving the condition most effective to reduce the deformations and to improve the surface qualities for most structures. In order to cut down the number of experimental tests, the Taguchi method was used in this research. An experimental scheme was designed by Minitab software based on *L*_27_ orthogonal array of Taguchi technique. It contains 27 rows that corresponds to 27 experimental runs with 26 degrees of freedom. The control factors were set differently for the support A and B. For the support A, the input or control factors are *G*_upper_ (Figure 3b, the gap between the upper surfaces of cuboids and the bottom surface of the overhang A), *H*_cuboid_ (the height of the cuboids), *G*_lower_ (the gap between the bottom surfaces of the cuboids and the upper surface of the base), and *D*_hatch_ (the distance between two adjacent and parallel walls of support structures) kept at three levels. For the support B, except for the same setting of *G*_upper_, *H*_cuboid_, *G*_lower_, and *D*_hatch_, the input or control factors also include *L*_top_ (the top length of the teeth of support structures) and *G*_teeth_ (the gap between every two adjacent teeth) kept at three levels. The values at different levels of the geometric parameters above are listed in Table 3. In this work, *H*_teeth_ (the height of the teeth) and *L*_base_ (the base length of the teeth of support structures) were set equal to *G*_upper_ and *L*_top_ respectively. Considering that the cuboids are built directly on powder, *G*_inner_ (the inner gap between the cuboids and thin walls) was set as 0 mm to prevent the deformation of the cuboids. The complete set of experiments is shown in Table 4.

In Taguchi methodology, the signal-to-noise ratio (*S*/*N*) can be applied to predict the optimal parametric combinations [20]. The experiment objective in this work was to optimize the support structure parameters to obtain an improvement of the bottom surface roughness and a reduction of the deformation. Therefore, the-smaller-the-better characteristic form was selected and the formula of *S*/*N* ratio is as follows:

(1) $$\eta =-10\;\log_{10}\left[\left(1/n\right)\overset{n}{\underset{i=1}{\sum }}y_{i}^{2}\right]$$

The η and *n* respectively represent the *S*/*N* ratio calculated from observed values and the number of repetition of each experiment and the *y_i_* is the experimental observed value of the *i*th experiment. Analysis of variance (ANOVA) was then used to discuss the relatively important factor of all controllable factors on the roughness and the deformation of the samples built by SLM and also to determine which one has the most significant effect [30].

For a further confirmation of the obtained influence rule, additional three sets of specimens with different gaps of 0.24 mm, 0.48 mm and 0.72 mm are designed for tensile tests, morphology and microstructure inspections. Each set contains four specimens, three for tensile tests and the other for morphology and microstructure inspections. The cross sections of the specimens are designed as square whose side length is 8 mm. The gaps are fully filled by same teeth structures whose *D*_hatch_ is 0.5 mm, *L*_top_ is 0.6 mm and *G*_teeth_ is 0.6 mm.

#### 3.2.3. Evaluation of Deformation and Surface Roughness

Different geometric designs of support structures result in deformations at different levels. In this research, the warp of the specimens were divided into four levels (Table 4): no warp, warp occurring between the cuboids and the bases, slight warp occurring at the teeth between the bottom surface of sample and the upper surface of cuboids and critical warp that disturbs the building process and occurs at the teeth as well.

Several distinct techniques, such as focus variation, tactile profile measurement, confocal laser scanning microscope and fringe projection technique, were used to detect the surface roughness on additive manufactured parts in the research in [31]. The results show the fact that different measuring methods used for same parts will achieve different values of average roughness. Although both the arithmetic average roughness *R*_a_ and the maximum height roughness *R*_z_ are very easily influenced by outliers and do not best qualify for characterizing surface roughness for powder bed manufactured parts, the values of *R*_a_ allow a narrower distribution than those of *R*_z_ because the roughness peaks and valleys could compensate each other out [31]. In this work, the Multi Function Tribometer (Rtec Instruments, San Jose, CA, USA) equipped with a white-light spectral interferometer equipment was used to acquire the roughness values of Ra based on the technique of focus variation. Repeated measurements for 9 times were conducted for each specimen and their means were regarded as the final values of *R*_a_.

## 4. Results and Discussion

### 4.1. Effect of Supports on the Deformation

**Figure 7:**
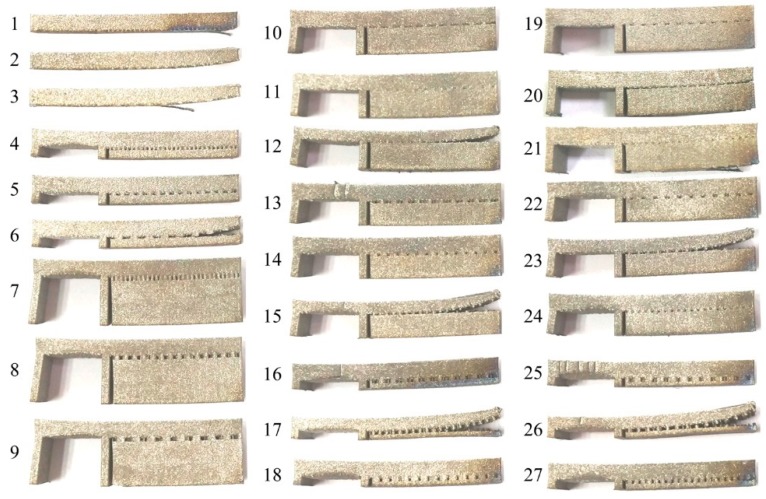
Specimens cut from the build platform by wire electrical discharge machining.

Figure 7 shows the specimens cut from the build platform by wire electrical discharge machining. The distortions of the left fixed side of the overhang A indicate that the residual stresses are very large in the SLMed parts. Therefore, the metal parts fabricated using powder bed additive manufacturing technology usually need heat treatment to dissipate their residual stresses [32].

The average *S*/*N* ratio for warp at each level of each factor are calculated according to the results in Table 4 and shown in Table 5. The Delta statistic which is the maximum minus the minimum average for each factor can compare the relative magnitude of effects. From Table 5, it can be concluded by ranking the Delta statistics that the factor with the greatest impact on the *S*/*N*_W_ ratio is *D*_hatch_ followed by *L*_top_, *G*_teeth_, *G*_upper_, *H*_cuboid_, and *G*_lower_. That is to say, the factors (*D*_hatch_, *L*_top_, and *G*_teeth_) related to the geometry of teeth generally have greater effect on the deformation of part than those (*G*_upper_, *H*_cuboid_, and *G*_lower_) related to the geometry of cuboids for heat dissipation. Therefore, the geometric design of teeth, especially *D*_hatch_, should be primarily considered. This conclusion is determined again by the results of ANOVA as shown in Table 6. The lower *p* value shows the more statistical significance of the effect of factor [33]. The *p* values of *D*_hatch_ (0.001), *L*_top_ (0.006), and *G*_teeth_ (0.007) are much lower than those of *G*_upper_ (0.067), *H*_cuboid_ (0.070), and *G*_lower_ (0.129).

**Figure 8:**
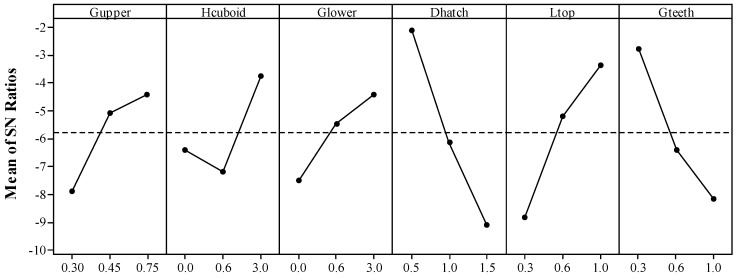
Main effects plot for *S*/*N*_W_ ratio.

As shown in Figure 8, the main effects plot for *S*/*N*_W_ ratio reflects clearly that the warp grade increases rapidly with increasing the *D*_hatch_ from 0.5 mm to 1.5 mm, *G*_teeth_ from 0.3 mm to 1.0 mm and decreasing the *L*_top_ from 0.3 mm to 1.0 mm. These three teeth geometric parameters directly influence the contact area between the support and the solid part. The *D*_hatch_ value expresses the mesh size of the support structure, which defines the whole length of support walls in a specified downward surface. When the whole length of support walls is fixed, the bigger *L*_top_ increases the contact area while the bigger *G*_teeth_ decreases the contact area. Obviously, the connection between the support and the solid part can be enhanced by increasing their contact area. Finite element analysis also shows that unequally spaced support structures change the heat dissipation pattern in the thin plate leading to thermal distortions [15].

The geometric parameters related to the cuboids such as *G*_upper_ and *H*_cuboid_ also show significant effects which are barely slighter than those of the geometric parameters related to the teeth. The results in Figure 8 show that the cuboids also affects the heat dissipation pattern which can influence the formation of the part in SLM. The warp of the overhang B can be reduced by increasing the height of the cuboid. However, the deformations of the specimens with height of 0.6 mm would slightly deteriorate comparing to those of the specimens without adding cuboids (*H*_cuboid_ = 0 mm) because the thin solid pieces with weaker bending resistance may influence the following fabrication of the teeth and the solid parts. It may be improved by fragmenting the support structure including the cuboids. When the value of *G*_lower_ goes up, the deformation magnitude also goes up since the larger gap between the cuboids and the platform lowers the effective heat conductivity. As the value of *G*_upper_ increases from 0.30 mm to 0.75 mm, it is easier to form the overhang. In this work, the value of *G*_upper_ is equal to the value of *H*_teeth_. However, the formation of part will be deteriorated with the increase of *H*_teeth_ according to the research in [20] where there’re no cuboids added in the block support. In the following, an experiment with a single factor variable validated the similar rule in this work about the effect of *G*_upper_ on the deformation. The tensile properties, morphologies and microstructures of part and its support structure are also analyzed.

### 4.2. Tensile Property, Morphology and Microstructure

**Figure 9:**
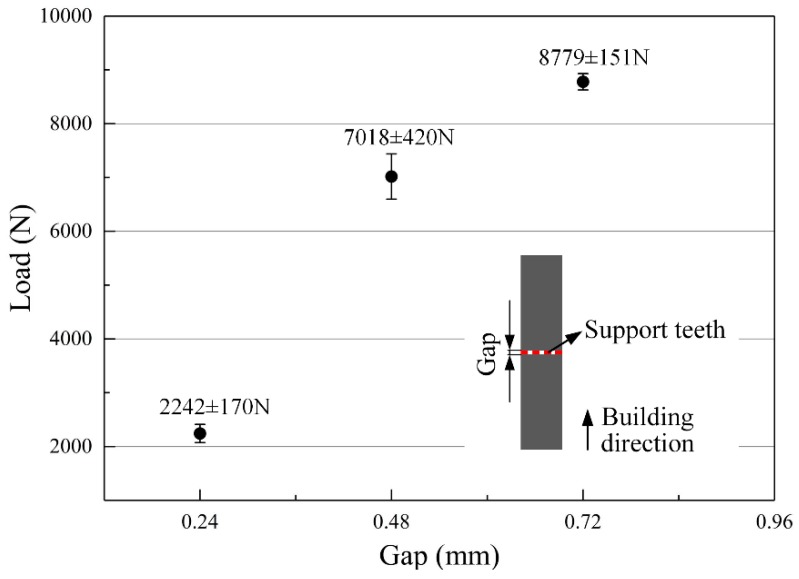
Tensile test results of samples with different gaps.

As shown in Figure 9, the tensile results show a stressed consistency with the test results acquired by the Taguchi method. The maximum tensile load after broken of specimen decreases from 8779 N to 7018 N and then sharply to 2242 N when the gap decreases from 0.72 mm to 0.48 mm and then to 0.24 mm. The acquired teeth thicknesses by measurements are 125 µm on average in all tensile specimens, which indicates that the total cross-section areas of the tensile specimens are approximately equal. Therefore, it can be concluded that the tensile strength of the support teeth is reduced approximately from 495 MPa to 396 MPa and then to 127 MPa when the gap decreases from 0.72 mm to 0.48 mm and then to 0.24 mm. Therefore, it is easier to form the overhang when the gap between the cuboids and the overhang goes up. Certainly, it should be noted that the *G*_upper_ cannot be too small. Otherwise, the cuboids will be connected to the solid part to induce a higher labor intensity of removing the support structures form the part.

**Figure 10:**
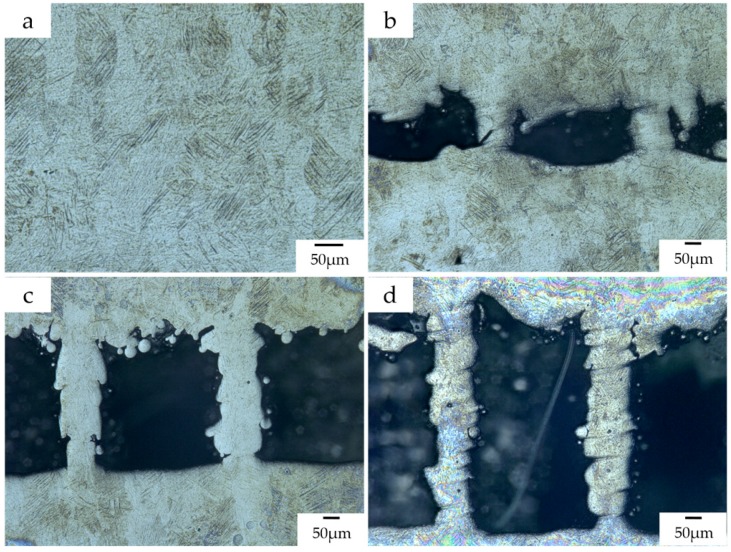
(**a**) The microstructure of the main body of the tensile specimens; the microstructures of the support teeth of the tensile specimens whose designed gap is (**b**) 0.24 mm, (**c**) 0.48 mm, and (**d**) 0.72 mm.

The microstructure of Ti–6Al–4V typically consists of a mixture of α and β phases at room temperature [25]. The α phase will transform to β phase with increasing the temperature during the radiation by the laser beam. The cooling rate primarily affects the microstructural evolution of Ti–6Al–4V and the transformation of Ti–6Al–4V from β phase into the needle-like martensite (α’) occurs at high cooling rates (greater than 410 °C/s) [34]. The transformation to martensite α’ can be ensured because the cooling rate is much higher than 410 °C/s after the laser beam moves away in the SLM process [35]. Therefore, as shown in Figure 10a, columnar β grains that consist primarily of acicular martensite (α’) generates in the main body of the tensile specimens in the vertical plane. Similarly, a small amount of martensite α’ can also be seen in the support teeth of the tensile specimens.

**Figure 11:**
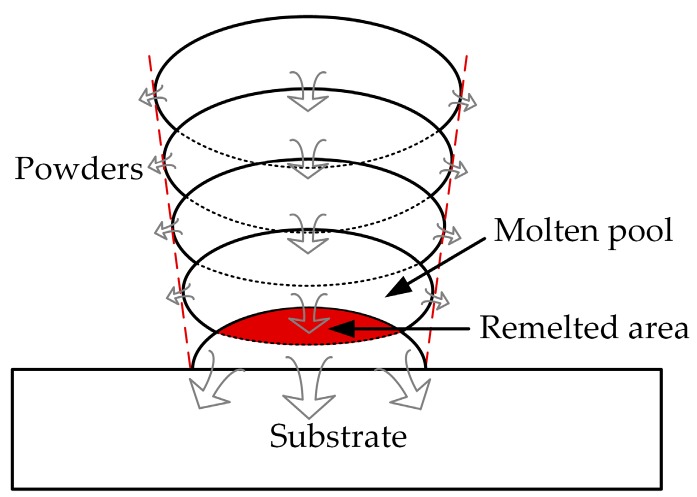
Mechanism of heat dissipation and overlap profiles of single tracks (support structures) in multiple layers.

On the other hand, it can be found from Figure 10b–d that the thickness of support teeth is not stable along the building direction and increases as the layer goes up. It results in a poor surface roughness of the support as the main body of the specimen. It may be resulted from the instability of the molten pool and the powder sticking effect. Meanwhile, as shown in Figure 11, the molten pool widens with deposit height and the remelted area of each layer caused by deposition of the succeeding layer also increases layer-by-layer [36]. This is because the condition of heat dissipation becomes worse. In the bottom region of the support structure closer to the substrate, the heat can be rapidly transported to the substrate due to the higher heat conductivity of the solid part. When the layer number increases, the heat in the molten pool cannot be dissipated in time because the effective heat conductivity of powder bed is much lower. This also clarifies that the positions where fractures occur during the tensile tests are mostly in the bottom regions of the support teeth. Therefore, it is worthwhile to design the teeth structure as trapezoid with longer bottom side.

### 4.3. Effect of Supports on the Roughness

As shown in Table 7, the response table presents the mean *S*/*N*_R_ ratio at each level of *G*_upper_, *H*_cuboid_, *G*_lower_, and *D*_hatch_ based on the results in Table 4. It can be concluded by ranking the Delta statistics that the factor with the greatest impact on the *S*/*N*_R_ ratio is *G*_upper_, followed by *G*_lower_, *H*_cuboid_, and *D*_hatch_. The *p*-values in Table 8 acquired by ANOVA show that the effect of *G*_upper_ is much more significant than those of *G*_lower_, *H*_cuboid_, and *D*_hatch_. The factor *G*_upper_ contributes 65.80% of the total variance and the factor *D*_hatch_ almost has no influence on the bottom surface quality of the overhang A.

**Figure 12:**
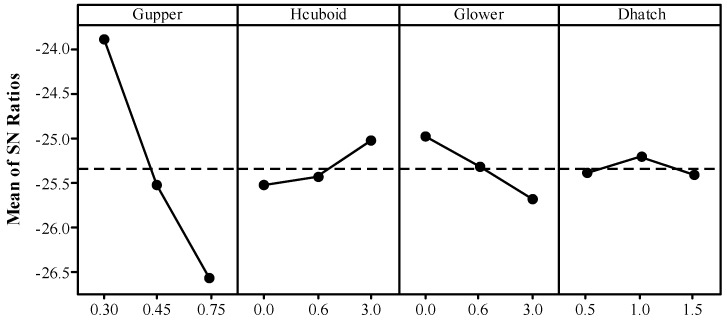
Main effects plot for *S*/*N*_R_ ratio.

**Figure 13:**
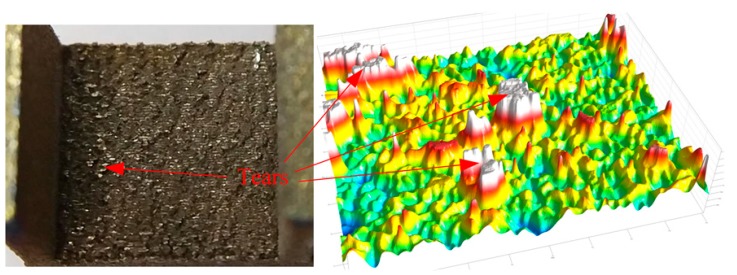
Morphology of the bottom surface of the overhang A in specimen 20.

The main effects plot for *S*/*N*_R_ ratio (Figure 12) clearly indicated that the bottom surface quality of the overhang A will be improved by the reduced *G*_upper_. It is also demonstrated by the fact, as shown in Figure 10b–d, that the amount of powder sticking to the bottom surface when the gap is 0.48 mm or 0.72 mm is much larger than that when the gap is 0.24 mm. However, the *G*_upper_ should be properly selected. If it is too small, the cuboids will be connected to the solid part, which results in an increase of the labor intensity in removing the support structures form the part. Meanwhile, the surface roughness will be influenced seriously. For example, as shown in Figure 13, a little of tears has been produced after removing the support structures from the part. In addition, increasing the *H*_cuboid_ and reducing the *G*_lower_ may also improve the bottom surface quality of the overhang.

## 5. Example of Support Addition

**Figure 14:**
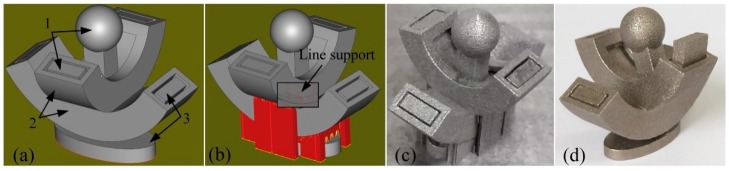
An example of non-assembly mechanical structure with two degrees of freedom: (**a**) model; (**b**) support generation; (**c**) as-built; and (**d**) as-built (mobilizable).

Considered as a case of complicated shape, a non-assembly mechanical structure with two rotational degrees of freedom (Figure 14) is designed to be fabricated by SLM. It is composed by three members. Although the AM technology provides a greater freedom of part design in theory, the manufacturability design of AM component must be given equal value to the functionality design during the design process. At present, there are still many limitations in SLM, such as limited size, minimum wall thickness, minimum hole diameter, minimum clearance, and maximum inclination. Especially, the clearance is critical during SLM fabrication of non-assembly mechanisms [37]. Based on the above experimental results, the clearances in kinematic pairs of this non-assembly structure were primarily determined as 0.30 mm for the complete separation of kinematic pairs.

After finishing the design of product and transforming it as stereolithography (STL) format, it is ought to be orientated properly in the build platform. Adjusting the part’s orientation can alter the inclined angles of overhanging surfaces to minimize the amount of support structures as much as possible [20]. Meanwhile, the removability of support and the requirement in surface roughness of the part must be taken into consideration during optimizing part’s orientation. In as-built SLMed parts, the morphologies of upward surfaces are generally much smoother than those of downward surfaces due to the effects of different thermal environments. Therefore, the surfaces with higher requirements should be orientated into upward surfaces.

If there are still overhanging structures that need to be supported, the type of support should be selected at first based on the size and shape of the supported surface area. As depicted in Figure 14b, line support was chosen to anchor the lowest part of the member 1 for an easier removal of support structures. At the same time, the small clearance between the kinematic pairs will make the teeth of the line support weak to be more easily removed from the gap and improve the surface roughness of the kinematic pairs. When generating support structures under the downward surfaces, additive solids as cuboids in this research added into conventional block support can improve the heat conduction efficiency of support structure and then decrease the deformation of the part.

## 6. Conclusions

Improper geometric design of the support structures may result in a sharp deterioration in the surface quality and even failure of manufacture. In this research, an unconventional block support structure whose interspaces are fully or partially filled by solid cuboids for heat dissipation was used to anchor the designed specimens with some overhanging structures. The Taguchi method was used to analyze the effect of the geometric design of this support on the part’s deformation and surface roughness. The main conclusions are as follows:

- Solid pieces or cuboids as support structures can reduce the deformation and then increase the forming property of SLMed overhanging structure through improving the heat dissipation condition;
- The teeth connecting supports and part are more effective than the added solid cuboids at resisting the deformation of part. Therefore, when designing the support structure, the parameters related to the tooth geometry should be mainly considered;
- The strength of the support teeth can be weakened approximately from 495 MPa to 396 MPa and then to 127 MPa by narrowing the gap between the cuboids and the overhang from 0.72 mm to 0.48 mm and then to 0.24 mm. Similar to the main body of the tensile specimen, small amount of martensite α’ can also be found in the support teeth. The thickness of support teeth is unstable along the building direction and increases layer-by-layer;
- The distance between every two adjacent walls of support and the gap between the cuboids and the overhang, respectively, most influence the part’s deformation and surface quality. As the gap between the cuboids and the overhang decreases, the bottom surface of part has a better quality.
- Absolutely, the gap between the cuboids and the overhang cannot be too small. A gap of 0.3 mm is preferred to form a non-assembly mechanical structure in this work.

## Figures and Tables

**Table 1:** Chemical compositions of used Ti–6Al–4V powder.

Elements	Al	V	Fe	O	C	N	H	Ti
Content (wt. %)	5.5–6.75	3.5–4.5	<0.3	<0.2	<0.08	<0.05	<0.015	Balance

**Table 2:** Process parameters of used Ti-6Al-4V powders.

Process Parameter	Value
Laser power	170 W
Scanning speed	1250 mm/s
Hatch distance	100 µm
Spot diameter	100 µm
Layer thickness	30 µm
Preheating temperature	35 °C
Atmosphere	Ar (Oxygen level <0.1%)

**Table 3:** Support parameter values.

Level	*G*_upper_ (mm)	*H*_cuboid_ (mm)	*G*_lower_ (mm)	*D*_hatch_ (mm)	*L*_top_ (mm)	*G*_teeth_ (mm)
1	0.30	0	0	0.5	0.3	0.3
2	0.45	0.6	0.6	1.0	0.6	0.6
3	0.75	3.0	3.0	1.5	1.0	1.0

**Table 4:** Design of experiments and results.

N	*G*_upper_ (mm)	*H*_cuboid_ (mm)	*G*_lower_ (mm)	*D*_hatch_ (mm)	*L*_top_ (mm)	*G*_teeth_ (mm)	Warp (–)	Roughness (µm)	*S*/*N*_W_ (dB)	*S*/*N*_R_ (dB)
1	0.3	0	0	0.5	0.3	0.3	3	16.9	−9.54	−24.56
2	0.3	0	0	1	0.6	0.6	4	17.3	−12.04	−24.76
3	0.3	0	0	1.5	1	1	4	17.1	−12.04	−24.66
4	0.45	0.6	0.6	0.5	0.3	0.3	1	20.9	0.00	−26.40
5	0.45	0.6	0.6	1	0.6	0.6	3	21.2	−9.54	−26.53
6	0.45	0.6	0.6	1.5	1	1	4	21.7	−12.04	−26.73
7	0.75	3	3	0.5	0.3	0.3	1	23.4	0.00	−27.38
8	0.75	3	3	1	0.6	0.6	1	24.1	0.00	−27.64
9	0.75	3	3	1.5	1	1	2	23.8	−6.02	−27.53
10	0.3	0.6	3	0.5	0.6	1	3	16.5	−9.54	−24.35
11	0.3	0.6	3	1	1	0.3	1	16.1	0.00	−24.14
12	0.3	0.6	3	1.5	0.3	0.6	4	15.8	−12.04	−23.97
13	0.45	3	0	0.5	0.6	1	1	17.6	0.00	−24.91
14	0.45	3	0	1	1	0.3	1	16.8	0.00	−24.51
15	0.45	3	0	1.5	0.3	0.6	4	17.1	−12.04	−24.66
16	0.75	0	0.6	0.5	0.6	1	1	22.3	0.00	−26.97
17	0.75	0	0.6	1	1	0.3	1	20.1	0.00	−26.06
18	0.75	0	0.6	1.5	0.3	0.6	4	21.5	−12.04	−26.65
19	0.3	3	0.6	0.5	1	0.6	1	13.8	0.00	−22.80
20	0.3	3	0.6	1	0.3	1	3	13.3	−9.54	−22.48
21	0.3	3	0.6	1.5	0.6	0.3	2	14.7	−6.02	−23.35
22	0.45	0	3	0.5	1	0.6	1	18.2	0.00	−25.20
23	0.45	0	3	1	0.3	1	4	18.5	−12.04	−25.34
24	0.45	0	3	1.5	0.6	0.3	1	19	0.00	−25.58
25	0.75	0.6	0	0.5	1	0.6	1	19.8	0.00	−25.93
26	0.75	0.6	0	1	0.3	1	4	18.6	−12.04	−25.39
27	0.75	0.6	0	1.5	0.6	0.3	3	19	−9.54	−25.58
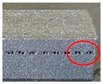 Warp 1			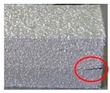 Warp 2			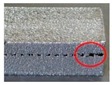 Warp 3		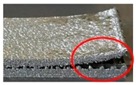 Warp 4		

**Table 5:** Average *S*/*N* ratio for warp.

Level	*G*_upper_	*H*_cuboid_	*G*_lower_	*D*_hatch_	*L*_top_	*G*_teeth_
1	−7.863	−6.412	−7.472	−2.121	−8.810	−2.789
2	−5.074	−7.195	−5.465	−6.134	−5.188	−6.412
3	−4.405	−3.736	−4.405	−9.088	−3.345	−8.141
Delta	3.458	3.458	3.067	6.967	5.465	5.352
Rank	4.5	4.5	6	1	2	3

**Table 6:** ANOVA for warp. *R*^2^ = 83.6%.

Source	DOF	Seq SS	MS	*F*	*p*	Contribution (%)
*G*_upper_	2	60.57	30.284	3.30	0.067	7.71
*H*_cuboid_	2	59.20	29.600	3.23	0.070	7.54
*G*_lower_	2	43.68	21.839	2.38	0.129	5.56
*D*_hatch_	2	220.12	110.062	12.00	0.001	28.03
*L*_top_	2	139.16	69.582	7.59	0.006	17.72
*G*_teeth_	2	134.26	67.128	7.32	0.007	17.09
Error	14	128.43	9.173	–	–	16.35
Total	26	785.42	–	–	–	100

**Table 7:** Average *S*/*N* ratio for Roughness (*R*_a_).

Level	*G*_upper_	*H*_cuboid_	*G*_lower_	*D*_hatch_
1	−23.90	−25.53	−24.99	−25.39
2	−25.54	−25.45	−25.33	−25.21
3	−26.57	−25.03	−25.68	−25.41
Delta	2.67	0.50	0.69	0.21
Rank	1	3	2	4

**Table 8:** ANOVA for Roughness (*R*_a_). *R*^2^ = 73.0%.

Source	DOF	Seq SS	MS	*F*	*p*	Contribution (%)
*G*_upper_	2	32.7634	16.3817	22.06	0.000	65.80
*H*_cuboid_	2	1.3039	0.6519	0.88	0.433	2.62
*G*_lower_	2	2.1236	1.0618	1.43	0.265	4.27
*D*_hatch_	2	0.2305	0.1152	0.16	0.857	0.46
Error	18	13.3696	0.7428	–	–	26.85
Total	26	49.7911	–	–	–	100
